# The effects of high shear rates on the average hydrodynamic diameter measured in biomimetic HIV Gag virus-like particle dispersions

**DOI:** 10.3389/fbioe.2024.1367405

**Published:** 2024-05-27

**Authors:** Tobias Wolf, Kerim Kadir Calisan, Jörn Stitz, Stéphan Barbe

**Affiliations:** ^1^ Research Group Medical Biotechnology and Bioengineering, Faculty of Applied Natural Sciences, TH Köln—University of Applied Sciences, Leverkusen, Germany; ^2^ Institue of Technical Chemistry, Leibniz University Hannover, Hannover, Germany

**Keywords:** HIV vaccines, virus-like particles, HIV Gag virus-like particles, mosaic virus-like particles, shear rate stability, hydrodynamic diameter, shear-induced aggregation

## Abstract

HIV Gag virus-like particles (HIV Gag VLPs) are promising HIV vaccine candidates. In the literature, they are often described as shear-sensitive particles, and authors usually recommend the operation of tangential flow filtration (TFF) gently at shear rates below 4,000 s^−1^ to 6,000 s^−1^. This in turn poses a severe limitation to the performance of TFF-mediated concentration of VLPs, which would be substantially enhanced by working at higher shear rates. To our knowledge, studies examining the shear sensitivity of HIV Gag VLPs and providing detailed information and evidence for the fragility of these particles have not been conducted yet. Thus, we investigated the effect of high shear rates on the colloidal stability of mosaic VLPs (Mos-VLPs) as relevant examples for HIV Gag VLPs. For this purpose, Mos-VLPs were exposed to different shear rates ranging from 3,395 s^−1^ to 22, 365 s^−1^ for 2 h. The average hydrodynamic diameter (AHD) and the polydispersity index (PDI) of the associated particle size distribution were used as stability indicators and measured after the treatment and during storage through dynamic light scattering. At high shear rates, we observed an increase in both AHD and PDI during the storage of HIV *Mos1.Gag* VLPs (bVLP—without envelope proteins) and *Mos1.Gag + Mos2S.Env* VLPs (eVLP—with envelope proteins). eVLPs exhibited higher colloidal stability than bVLPs, and we discuss the potential stabilizing role of envelope proteins. We finally demonstrated that the dispersion medium also has a considerable impact on the stability of Mos-VLPs.

## 1 Introduction

An ideal vaccine to fight HIV infection would elicit potent cellular and humoral immune responses. The extreme genetic variability of HIV-1 constitutes the highest hurdle in the development of a vaccine (Gaschen et al., 2002; Ndung’u and Weiss, 2012). Upon infection after vaccination, an elicited strong *CD8* ^*+*^ cytotoxic T-cell response should reduce the number of infected cells and suppress the viremia and virus burden (Kiepiela et al., 2007; Janes et al., 2017; Collins et al., 2020). Induced broadly neutralizing antibodies directed against neutralization-sensitive epitopes present in the viral envelope glycoproteins (Env) should inhibit virus cell entry (Zolla-Pazner et al., 2014; Caskey et al., 2017; Ding et al., 2021). In an attempt to improve the coverage of potential T- and B-cell epitopes stemming from a range of HIV variants, synthetically shuffled epitope sequences were employed to form the so-called mosaic (Mos) HIV antigens (Fischer et al., 2007; Barouch et al., 2010). Such Mos antigens and their cognate coding sequences (Baden et al., 2020; Langedijk et al., 2019; Langedijk et al., 2021) were components of the tetravalent vaccine *Ad26.Mos4.* HIV, which reached phase III in the clinical trial Mosaico (NCT03964415) but was unfortunately prematurely terminated as the achieved efficacies failed to reach the > 50% target. However, we recently demonstrated that co-expression of both *Mos.Gag* and a *Mos.En*v-encoding sequences results in virus protein synthesis, mediating the formation of virus-like particles (VLPs) decorated with Env proteins and exposing broadly neutralizing-sensitive epitopes required to elicit broadly neutralizing antibodies in vaccines (Rosengarten et al., 2022). VLPs are efficiently taken up by antigen-presenting cells that subsequently cross-present viral epitopes, and the target antigens displayed on the surface of VLPs facilitate cross-linking of B-cell receptors and VLPs and can traffic into lymph nodes, mediating the efficient stimulation of reactive B and T cells (Bachmann et al., 1993; Tsunetsugu-Yokota et al., 2003; Zhang et al., 2009; Bachmann and Jennings, 2010; Temchura et al., 2014; Slütter and Jiskoot, 2016). Consequently, VLPs are favorable vaccine components, capitalizing on their potent induction of cellular and humoral immunity (Paliard et al., 2000; Garrone et al., 2011; Nabi et al., 2013; Goepfert et al., 2014; Pitoiset et al., 2017; Perdiguero et al., 2019; Zhang et al., 2021). The stimulation of an immune response depends on the individual and intact VLPs, which are complex nanoparticles. Consequently, the loss of VLP integrity or their aggregation significantly impairs the efficacy of the vaccine, leading to a reduction in immunogenicity (Deng, 2018; Mohsen et al., 2018; Tariq et al., 2021; Tarrés-Freixas et al., 2023). Hence, Mos-VLPs may prove to be potent vaccine components in future vaccine development programs, warranting further testing in animal models. This led us to examine the physical characteristics of Mos-VLPs in detail to prepare for their production, purification, and concentration at larger scales.

It is a common practice to implement tangential flow filtration (TFF) as a unit operation for concentration purposes in the development of HIV vaccines based on HIV Gag VLPs (Zhang et al., 2009; Negrete et al., 2014; Helgers et al., 2022a). This highly scalable technique is well-established for vaccine development, and the upscaling strategy mainly relies on an increase in the membrane surface area (an increase in the module size), while the set flow conditions are similar to the ones optimized during the development phase. TFF involves forced liquid flow in thin hollow fibers, which is associated with considerable wall shear stress. HIV Gag VLPs have been often described as shear-sensitive nanoparticles, and authors recommend operating TFF gently at shear rate values lower than 4,000 s^−1^ to 6,000 s^−1^ (Negrete et al., 2014; Helgers et al., 2022b; Hengelbrock et al., 2022). It is well-known that mass transfer during the TFF treatment of such particles is limited by the formation of a particle polarization layer near the membrane surface, whose thickness and the resulting flow resistance can be minimized by working at high shear rates (Schock, 1985; Moleirinho et al., 2020; Wolf et al., 2022). We recently proposed a numerical approach to the study of HIV Gag VLPs TFF based on the friction model developed by Schock (1985) (Wolf et al., 2022). According to this model, the stability of the particle polarization layer mainly depends on the applied shear rate and the static coefficient of friction of HIV Mos-VLPs. Extrapolations of our model suggested that TFF performance can be substantially increased by applying shear rate values higher than 4,000 s^−1^ to 6,000 s^−1^.

We could not find any studies of the shear sensitivity of HIV Gag VLPs, which could provide detailed information and evidence of the fragility of these particles. Although high shear stress often causes the aggregation of VLPs (Cruz et al., 2000; Shi et al., 2005; Effio and Hubbuch, 2015), some authors mentioned that shear forces may also lead to the destruction and loss of integrity of VLPs (Nooraei et al., 2021; Gupta et al., 2023). Consequently, there is a substantial need for clarification in this field. In the present report, with regard to Mos-VLPs as relevant and representative examples of HIV Gag VLPs, we addressed the following questions:

• How does shear stress affect the colloidal stability of Mos-VLP dispersions?
• In what time interval can such destabilization phenomena be observed?
• Do Mos-antigens play any role in the colloidal stability of HIV Gag VLPs?
• Does the medium have an influence on the shear stability?
• What impact do the results of this investigation have on the TFF treatment of Mos-VLPs?

The average particle size and the polydispersity index (PDI) of the associated particle size distributions are suitable indicators of the aggregation and/or disruption phenomena in Mos-VLP dispersions. In this regard, dynamic light scattering is a well-established holistic method for the characterization of HIV Gag VLPs, which provides the average hydrodynamic diameter (AHD) of particles contained in a VLP dispersion (Lambricht et al., 2016; González-Domínguez et al., 2021; Lorenzo et al., 2023). AHD is equivalent to the diameter of a sphere, which exhibits the same translational diffusion coefficient as the investigated VLPs. We defined AHD and PDI as the characterization parameters for the colloidal stability of both VLP HIV *Mos1.Gag* VLPs (bVLP—without Env proteins) and *Mos1.Gag + Mos2S.Env* VLP (eVLP—with envelope proteins) dispersions exposed to different shear rates for different periods of time during their storage.

## 2 Materials and methods

### 2.1 Production and clarification bVLPs and eVLPs

HIV Mos1.Gag VLP (bVLPs) and Mos1.Gag + Mos2S.Env VLPs (eVLPs) were produced according to the protocol developed by Rosengarten et al. (2022) (Wolf et al., 2022). Briefly, the production of the HIV Mos-VLP was carried out in disposable shaker flasks with vent caps (Thermo Fisher Scientific™ Nalgene™, United States). As the producer cells, HEK293F suspension cells were used in FreeStyle 293 EM and were kindly provided by Rosengarten et al. (2022). The cells were inoculated with a starting concentration of 3×10^5^ cells/mL to 5×10^5^ cells/mL. Additionally, a constant selection pressure was applied by adding 15 μg mL^−1^ of puromycin (InvivoGen, Toulouse, France) for bVLPs and 10 μg mL^−1^ of puromycin and 200 μg mL^−1^ of hygromycin (InvivoGen, Toulouse, France) for eVLPs, respectively. The cultivation conditions for both were set to 37 °C, 8% CO_2_, and 135 min^−1^ (HERAcell 150i CO_2_ Incubator, Thermo Fisher Scientific, Waltham, Massachusetts, United States). After 3–4 d of inoculation, the cell suspension was centrifuged with 100 g for 5 min at 21°C using a Rotina 420R Centrifuge (Hettich, Germany), and the supernatant was filtered through a 0.45 µm PVDF filter (Carl Roth GmbH & Co. KG, Karlsruhe, Germany).

### 2.2 Replacement of the dispersion medium

We used ultracentrifugation to replace the medium from the cultivation (Freestyle 293 EM) with water. The cell-free supernatant (from Section 2.1) was therefore centrifuged at 25,000 min^−1^ (52,700 g to 113,000 g) for 2 h using a SW28 swing-out rotor and an Optima XE Centrifuge (Beckman Coulter, United States). The pellet was re-dispersed in the same volume of ultrapure water. Finally, the VLP water dispersion was filtered using a 0.22 µm PVDF filter to remove aggregates.

### 2.3 Average hydrodynamic diameter and polydispersity index

AHD and PDI were determined using a Zetasizer Nano ZS (Malvern Panalytical GmbH, Germany). The sample was placed in a quartz glass cuvette with a path length of 10 mm. For the measurements, 15 runs per sample were recorded, and each run took 10 s. The backscatter signal was detected at an angle of 173° at 25°C. The attenuator varied between 8 and 11. As for solvent properties for water, the refractive index was set to 1.33 and the viscosity to 0.8872 mPa s. The material properties were set as proteins with a refractive index of 1.45.

### 2.4 *ζ*-Potential

The Zetasizer Nano ZS (Malvern Panalytical GmbH, Germany) was also used for the determination of the *ζ*-potential. For this purpose, as a cuvette, a DTS1070 capillary zeta cell was used. The temperature was set to 25°C, and the material properties were set as described in Section 2.3. The count of runs was automatically generated using Zetasizer software.

### 2.5 Shear stability

A KrosFlo^®^ KR2i TFF System (Repligen Corporation, Waltham, Massachusetts 02453, United States) was used for the investigation of the effect of the shear rate on the colloidal stability of VLPs. To operate at a defined shear rate, we used a hollow fiber module with an effective length of 20 cm, a fiber inner diameter of 0.5 mm, and a fiber count of 6 (Repligen Corporation, Waltham, Massachusetts 02453, United States). The molecular weight cut-off (MWCO) was 70 kDa, which should not have an influence on the particle due to the large space between particle diameter and pore size (Wolf et al., 2022). Furthermore, the permeate outlet was closed during all experiments to avoid a particle polarization layer as well as cross-flow. The membrane material was modified PES. The tubing around the module had an inner diameter of 3.1 mm, and to minimize pulsation, dampers were installed before and after the peristaltic pump. The cleaning methods were the same as those reported by Wolf et al. (2022). The shear rate was adjusted *via* the volume flow rate, and the shear time was 2 h. After 2 h, the experiment stopped, and the remaining dispersion volume was aliquoted in 1.5 mL tubes and stored at 23°C.

## 3 Results and discussion

In this study, we investigated the effects of high shear rates on the AHD and PDI measured in bVLP and eVLP dispersions. A preliminary series of experiments with bVLP dispersions and shear rates up to 23,000 s^−1^ over long shear cycles (up to 72 h) showed that an increase in AHD and PDI was only observed after 3 days of storage at 5°C. A further preparative experiment was conducted with eVLPs exposed to a shear rate of 23,000 s^−1^ for 24 h. In this case, AHD and PDI started increasing after 7 days of storage at 5°C (Calisan, 2023). These observations suggest the existence of shear-induced aggregation phenomena and give rise to the assumption that such behavior is also present at lower shear rates. To confirm this hypothesis, AHD and PDI were measured after different storage times at room temperature in both bVLP and eVLP dispersions that were subjected to the shear rates listed in the heading of Table 1 for 2 h. The main results of this study are summarized in Table 1, and the corresponding data set is available as Supplementary Material (Supplementary Table S1). After shear treatment and for each shear rate, the resulting treated dispersion was pooled and aliquoted in 1.5 mL tubes. Subsequently, one aliquot per shear rate was then characterized using DLS (measurement of AHD and PDI). With Reynolds numbers far below 2,300, the flow regime in the hollow fibers was laminar in all experiments. Overall, no decrease in AHD or PDI was observed in this study, which could provide evidence for particle destruction or loss of VLP integrity, which is crucial for their vaccine application (Deng, 2018; Mohsen et al., 2018; Tariq et al., 2021). At this stage, we questioned the ability of the DLS equipment to detect the destruction of the VLPs. To verify this, we performed a series of experiments in which the VLPs were exposed to different surfactant concentrations (Triton X-100). We observed a decrease in AHD over time resulting from the destruction of the particles.

**TABLE 1:** Results of the long-term stability of both VLPs for different shear rates and different dispersion media. We defined threshold AHD and PDI values of AHD > 250 nm and PDI > 0.25, respectively, and assumed that particle aggregation occurs when these values are exceeded. After shear treatment and for each shear rate, the resulting treated dispersion was pooled and aliquoted into 1.5 mL tubes. On each subsequent day, one VLP dispersion aliquot per shear rate was characterized by DLS (measurement of AHD and PDI).

**Volume flow rate** $\dot{V}$	/ml min^−1^	15	25	40	62	80	100
**Shear rate** *γ*	/s^−1^	3,395	5,659	9,054	14,034	18,108	22,635
**Velocity** *u*	/m s^−1^	0.21	0.35	0.57	0.88	1.13	1.41
**Reynolds number** Re	/1	119	198	317	491	634	792
bVLP	FreeStyle	5 d	2 d	3 d	3 d	3 d	2 d
	Water	> 14 d	> 14 d	> 14 d	> 14 d	6 d	10 d
eVLP	FreeStyle	> 14 d	> 14 d	> 14 d	> 14 d	2 d	2 d
	Water	> 14 d	> 14 d	> 14 d	> 14 d	> 14 d	> 14 d

**FIGURE 1:**
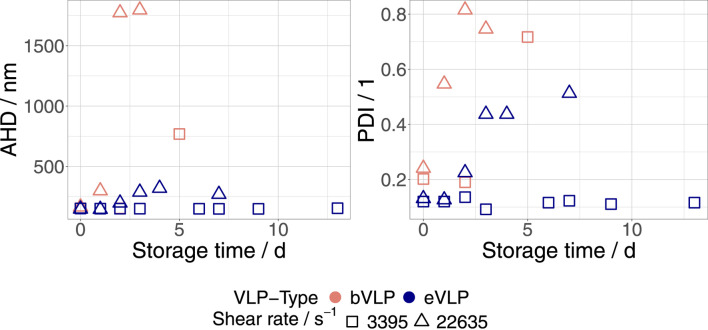
Progression of AHD and PDI measured in eVLP and bVLP FreeStyle 293 EM dispersions that were exposed to shear rates of 3,395 s^−1^ and 22,635 s^−1^, respectively. Samples were stored at 23°C.

Figure 1 depicts the progression of AHD and PDI measured in eVLP and bVLP FreeStyle 293 EM dispersions that were exposed to shear rates of 3,395 s^−1^ and 22,635 s^−1^ and stored at 23°C. An increase in AHD and PDI was observed for both VLP types within 2 days after treatment at the highest shear rate (22 365 s^−1^). This finding further supports the shear-induced aggregation hypothesis. We could not detect any changes in eVLP dispersions at the lowest applied shear rate (3,395 s^−1^). However, AHDs and PDIs measured in bVLP dispersions at this shear rate rose after 5 days over the threshold of 250 nm and 0.25, respectively. This difference in shear stability between eVLP and bVLP FreeStyle 293 EM dispersions was confirmed at intermediate shear rate values, as shown in Table 1. To facilitate data analysis, we defined the threshold AHD and PDI values of 250 nm and 0.25, respectively, and assumed that particle aggregation or destabilization occurs when these values are exceeded. After production and concentration, both VLP types are typically characterized by an AHD of 160 nm–170 nm and a PDI of 0.12–0.13 (Wolf et al. (2022)). The destabilization of eVLP FreeStyle dispersions was only detected at shear rate values higher than 14,034 s^−1^. As already observed in preliminary trials, eVLPs appear to be much more stable against shear constraints than bVLPs.

From a physico-chemical point of view, eVLPs only differ from bVLPs by the presence of envelope glycoproteins, Mos-antigens, on their surface, which seem to inhibit the shear-induced aggregation phenomena. A difference in surface charge density (SCD) between eVLPs and bVLPs is a reasonable explanation for this observation. In this regard, higher SCD values would result in higher repulsive forces between particles and consequently lead to an inhibition of the aggregation mechanisms. This property of nanoparticles is well known and often used to stabilize nanodispersions (Bouhaik et al., 2013). The *ζ*-potential is a measure for the SCD of nanoparticles that can be calculated from the measured electrophoretic mobility of a particle. We determined a *ζ*-potential of approximately 14 mV for both eVLPs and bVLP dispersed in FreeStyle 293 EM. Consequently, the higher stability of eVLPs cannot be explained by differences in SCD. The potential steric stabilization of envelope glycoproteins is another conceivable cause for this observation, but further experiments are needed to confirm the appropriateness of this explanation.

**FIGURE 2:**
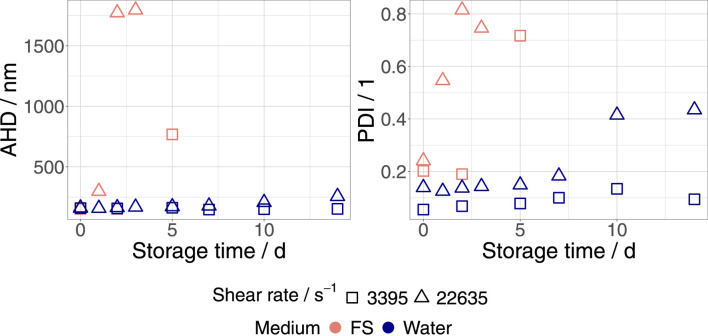
Progression of AHD and PDI in bVLPs dispersed in ultrapure water and FreeStyle 293 EM (FS) that were exposed to shear rates of 3,395 s^−1^ and 22,635 s^−1^, respectively. Samples were stored at 23°C.

Finally, we examined the influence of the dispersion medium on the shear stability of eVLPs and bVLPs. For this purpose, as described in Section 2.2, the FreeStyle 293 EM was substituted with ultrapure water. Ultrapure water and FreeStyle 293 EM have similar pH values of 7.0 and 7.0 to 7.6, respectively. In contrast to ultrapure water, FreeStyle 293 EM, as a chemically defined and protein-free culture medium, contains ions and polyelectrolytes, leading to higher ionic strength and conductivity (0.14 mS cm^−1^ to 0.26 mS cm^−1^ ultrapure water and 10.4 mS cm^−1^ to 11.2 mS cm^−1^ FreeStyle 293 EM). Figure 2 shows the evolution of AHD and PDI in bVLPs dispersed in ultrapure water and FreeStyle 293 EM that were exposed to shear rates of 3,395 s^−1^ and 22,635 s^−1^, respectively. The shear stability of bVLPs was dramatically enhanced by medium replacement. As shown in Table 1, the same stabilizing effect was also observed in eVLP water dispersions, where no aggregation could be detected. We determined a higher *ζ*-potential of approximately 30 mV for both eVLPs and bVLPs dispersed in ultrapure water. Consequently, this finding may be explained by an increase in the magnitude of repulsive forces between VLPs in ultrapure water.

## 4 Conclusion

For the first time, the present brief report provides a systematic approach to the shear stability of Mos-VLPs as relevant and representative examples for HIV Gag-based VLPs. The latter are currently considered promising HIV vaccine candidates. We showed that the exposition of eVLPs (HIV *Mos1.Gag + Mos2S.Env* VLPs) and bVLPs (HIV *Mos1.Gag* VLPs) to shear rates ranging from 3,395 s^−1^ to 22,635 s^−1^ leads to aggregation phenomena (increase in AHD and PDI) during particle storage at room temperature. We observed such destabilization processes within 2–10 days after the shear treatment. The eVLPs involved in this study had much higher shear stability (up to 14 034 s^−1^) than the bVLPs. This surprising difference cannot be explained in terms of SCD, and further experiments are needed to understand this finding. We could not find any indications of VLP disruption in this study.Replacing FreeStyle 293 EM with ultrapure water led to a substantial increase in the shear stability of both eVLPs (up to 22 635 s^−1^) and bVLPs (up to 14 034 s^−1^), and the increased repulsive forces between VLPs can explain this effect. Overall, Mos-VLPs exhibit higher shear stability than assumed in the literature, especially when they are dispersed in low-salinity media, and we think that operating the enhanced TFF treatment of Mos-VLPs at higher shear rates (up to 23,000 s^−1^) might be feasible. The high shear stability of eVLPs has a positive effect on the operating conditions of TFF, which can be carried out at shear rates of 10,000 s^−1^ to 14,000 s^−1^. According to the prediction of Wolf et al. (2022), the TFF concentration and purification of Mos-VLPs at shear rates of more than 4,000 s^−1^ to 6,000 s^−1^ can lead to a significant improvement in the filtration process, which can be completed in a shorter time and with a smaller filtration area. However, further antigenic characterizations and high-resolution images are needed to ensure that vaccine potency is not affected by shear constraints. As the Mos-VLP dispersions presented are primarily intended for the vaccination of humans, this study should be supplemented by an investigation of their colloidal stability in bodily fluids such as blood or plasma.

## Data Availability

The original contributions presented in the study are included in the article/Supplementary Material; further inquiries can be directed to the corresponding author.

## Abbreviations

AHD, average hydrodynamic diameter; bVLP, HIV Mos1.Gag VLP; Env, viral envelope glycoproteins; eVLP, HIV Mos1.Gag + Mos2S.Env VLP; Mos, mosaic; PDI, polydispersity index; SCD, surface charge density; TFF, tangential flow filtration; VLP, virus-like particle.
